# Structural insights into the cooperative remodeling of membranes by amphiphysin/BIN1

**DOI:** 10.1038/srep15452

**Published:** 2015-10-21

**Authors:** Julia Adam, Nirakar Basnet, Naoko Mizuno

**Affiliations:** 1 Cellular and Membrane Trafficking, Max Planck Institute of Biochemistry, Am Klopferspitz 18, D-82152 Martinsried, Germany

## Abstract

Amphiphysin2/BIN1 is a crescent-shaped N-BAR protein playing a key role in forming deeply invaginated tubes in muscle T-tubules. Amphiphysin2/BIN1 structurally stabilizes tubular formations in contrast to other N-BAR proteins involved in dynamic membrane scission processes; however, the molecular mechanism of the stabilizing effect is poorly understood. Using cryo-EM, we investigated the assembly of the amphiphysin/BIN1 on a membrane tube. We found that the N-BAR domains self-assemble on the membrane surface in a highly cooperative manner. Our biochemical assays and 3D reconstructions indicate that the N-terminal amphipathic helix H0 plays an important role in the initiation of the tube assembly and further in organizing BAR-mediated polymerization by locking adjacent N-BAR domains. Mutants that lack H0 or the tip portion, which is also involved in interactions of the neighboring BAR unit, lead to a disruption of the polymer organization, even though tubulation can still be observed. The regulatory region of amphiphysin/BIN1 including an SH3 domain does not have any apparent involvement in the polymer lattice. Our study indicates that the H0 helix and the BAR tip are necessary for efficient and organized self-assembly of amphiphysin/N-BAR.

Lipid bilayer membranes are essential components of a cell for the separation from the surrounding environment and for intracellular compartmentalization. Particular importance of the cellular membranes lies on their dynamic and flexible morphology, which is used for shaping diverse cellular components. This feature is essential for cellular trafficking such as vesicular transport or endocytosis. Specific membrane shapes are formed from flat surfaces by a coordinated deformation and remodeling^1^.

For shaping membranes several regulator proteins are involved^2^,^3^. An important example of such membrane curving proteins is the BAR (Bin/Amphiphysin/Rvs) domain superfamily^4^,^5^,^6^. The highly conserved BAR domains form variably shaped dimers, which are used as a mold to shape membranes. The BAR domains recognize membrane curvatures via electrostatic interactions between the positive charges on its curved surface and the negative charges of the membrane headgroups. This causes a membrane to bend according to the intrinsic curvature of the BAR dimers - a mechanism called “scaffolding”^7^,^8^,^9^. A sub-class of the BAR superfamiliy, N-BAR proteins contain an N-terminal amphipathic helix termed H0, presumably located at the edge of the concave surface of a crescent-shaped BAR dimer. Previously, *in vitro* biophysical experiments showed that the H0 helices are only structured in the presence of lipids and the H0 helices are embedded on one leaflet of the membrane surface with its amphipathic feature^10^,^11^,^12^,^13^, leading to the distortion of the membrane – a mechanism called “wedging”^14^,^15^,^16^,^17^. The crescent-shaped structure of N-BAR proteins has been well characterized by X-ray crystallography^4^,^7^, cryo-EM 3D reconstructions of another N-BAR protein endophilin^18^,^19^ and computational simulations^20^,^21^. These studies revealed that BAR proteins are polymerizing in a helical manner (lattice of spiraling rows) around the membrane tube, which is held together by BAR dimer-dimer interactions, however, the interaction with membranes including H0 is not well understood.

Amphiphysin is an N-BAR protein, involved in clathrin-mediated endocytosis (CME)^22^. It is thought to contribute to the dynamic curvature formation at the neck of budding vesicles during endocytosis by coordinating with other curvature forming proteins like clathrin, endophilin and dynamin^23^,^24^. While amphiphysin in CME is involved in the dynamic membrane deforming process, an isoform of amphiphysin, amphiphysin2/BIN1 is found as a component involved in the structural organization of muscle T-tubules^25^,^26^. T-tubules are deformed plasma membranes of muscle sarcolemma giving a platform for the excitation-contraction coupling machinery^27^. In Drosophila, amphiphysin is only implicated in T-tubule formation^28^,^29^, and defects of amphiphysin show a phenotype of viable, flightless flies with a major disorganization of T-tubules^26^. Myoblastic C2C12 cell analysis showed the induction of T-tubule-like structures upon amphiphysin/BIN1 expression^25^. Together with the finding that vesicles are remodeled into a tubular shape in the presence of amphiphysin/BIN1 *in vitro*^10^,^30^ and that the protein possesses a membrane curving BAR domain^10^, amphiphysin/BIN1 is thought to be responsible for tubulogenesis of T-tubules.

Amphiphysin/BIN1 consists of a BAR domain followed by a region with unknown structure and a SH3 (Src Homology 3) domain. The SH3 domain is thought to recruit the downstream interaction partner dynamin. Mutations of the human amphiphysin/BIN1, K35N located in H0, D151N, R154Q in the BAR domains and Q434X and K436X in the SH3 domain lead to a neuromuscular disorder called centronuclear myopathy (CNM)^31^,^32^,^33^,^34^ with various degrees of muscle weakness. CNM patients show a defect in the organization of the T-tubules, highlighting the importance of the formation of ordered T-tubules. *In vitro*, amphiphysin/BIN1 is able to transform liposome vesicles into narrow tubules^10^,^13^ like other BAR domain proteins^18^,^19^,^35^,^36^,^37^. However, until now it is still not understood what the structural organization of amphiphysin/BIN1 is, how it remodels membranes and what its implication in T-tubules formation is.

In this work, we present the structural basis of the amphiphysin/BIN1 membrane remodeling activity using *in vitro* reconstitution and cryo-EM. To assess the effect on tube formation, a series of Drosophila amphiphysin/BIN1 truncations were produced and their membrane interactions were tested. Amphiphysin/BIN1 self-assembles on membrane surfaces in a cooperative manner to remodel a vesicle to a tube. The N-terminal H0 helix is not necessary for the membrane remodeling activity but it is required for fast and rigid tube formation. The regulatory domains were not incorporated into the tube but protruding outwards. The cryo-EM 3D reconstructions of amphiphysin/BIN1 tubes revealed a unique assembly of amphiphysin/BIN1 BAR domains wrapping around the membrane to form a membrane tube. Amphiphysin/BIN1 is tightly packed with its one tip immersed into the lipid bilayer, while the other tip protrudes out from the tube. A rod-like density connecting adjacent BARs, likely the H0 helix, stabilizes the BAR dimer-dimer interactions. The size of the tube, which is determined by the arrangement of the BAR domains was not as variable as for the case of other N-BAR mediated tubes^18^ but local fluctuations were detected in which the rod-like density is no longer connecting the BAR units. Altogether, our analysis shows H0 is the key to efficiently arrange BAR dimers for an organized polymerization on a membrane surface.

## Results

### H0 helix facilitates efficient tube formation

**Figure 1:**
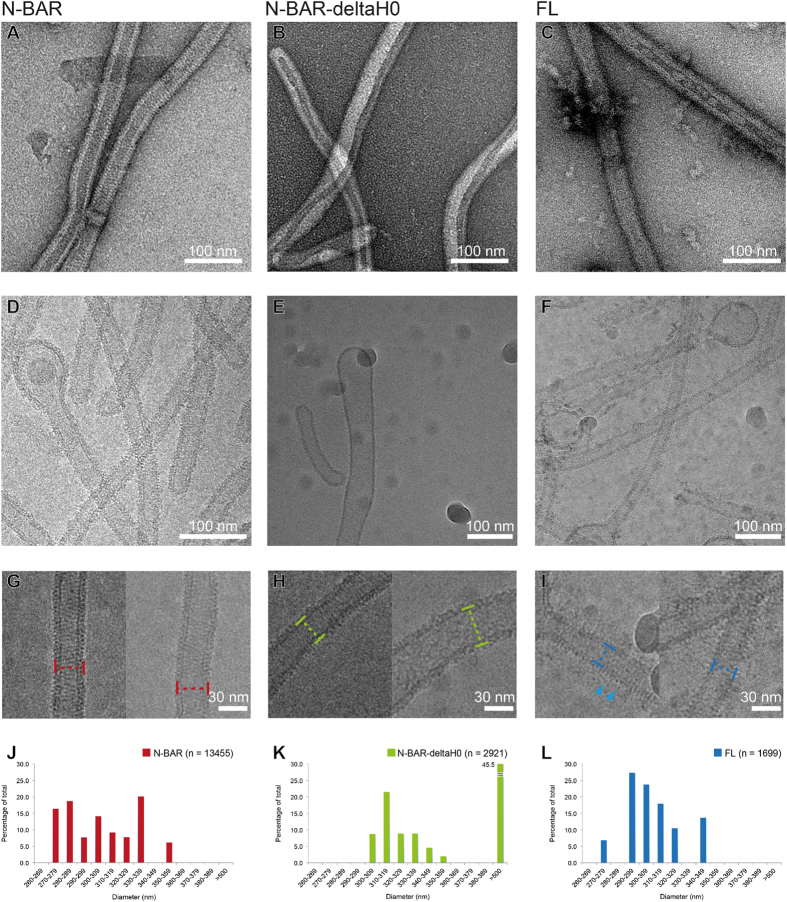
(**A**–**C**) Negative-stain EM images of tubes mediated by N-BAR (**A**), N-BAR-deltaH0 (**B**) and FL amphiphysin/BIN1(**C**). (**D**–**F**) Cryo-EM images of tubes mediated by N-BAR (**D**), N-BAR-deltaH0 (**E**) and FL **(F**). (**G–I**) Zoom-in views of N-BAR tubes (**G**), a thin N-BAR-deltaH0 tube (**H**, left) and a thick N-BAR-deltaH0 tube (**H**, right), and FL tubes (**I**). Markers show the definitions of the width at the outer membrane leaflet. In (**I**), needle-like densities around tubes are shown (arrowhead). (**J**–**L**) Histograms of the distributions of the width of the tubes mediated by N-BAR (**J**), N-BAR-deltaH0 (**K**) and FL (**L**).

Amphiphysin/BIN1 has been shown to remodel vesicles into tubes^10^,^13^,^30^, resembling the formation of T-tubules in muscle cells^25^. To explore the roles of the H0 helix, the BAR domain and the regulatory regions of amphiphysin/BIN1 in the context of their membrane interactions, we created amphiphysin/BIN1 fragments including full length amphiphysin/BIN1 (FL, residues 1-602), full length amphiphysin/BIN1 without the H0 helix (deltaH0, 27-602), only N-BAR domain (N-BAR, 1-244) and N-BAR domain without H0 (N-BAR-deltaH0, 31-244 or 27-247) and followed membrane-remodeling by monitoring light-scattering at 400 nm (Table S1A). In all cases, an increase in scattering was observed as proteins and liposomes were mixed. This increase corresponded to the tube formation judging from the corresponding negative stain EM observation (Table S1B, Table S1C, Fig. 1A–C). While the N-BAR liposome mixture showed an immediate increase in scattering, the degree of scattering increase was lower in the presence of amphiphysin/BIN1 lacking H0 (deltaH0, N-BAR-deltaH0) (Table S1A, green and blue) or FL (Fig. S1A, orange). Moreover, a gradual decrease of the scattering signal was observed for N-BAR and FL (Fig. S1A, red and orange), while the scattering stayed constant for the case of deltaH0 and N-BAR-deltaH0 (Fig. S1A, green and blue). Negative-stain EM images of the mixture after 30 min of incubation showed that stable tubes are still retained when BAR fragments are added to liposomes (Fig. S1C, “+N-BAR-deltaH0”), while many of the tubes turned into small vesicles (~300 Å in size) in the presence of the proteins containing H0 helices (N-BAR) (Fig. S1C, “+N-BAR”, red arrowhead). Occasionally we observed vesicles squeezed out from a tube (Fig. S1C, “+N-BAR”, 30 min). By comparing these observations, the effects of H0 are pinpointed 1) to promote the initiation of membrane remodeling and 2) to finally squeeze the remodeled tubes into vesicles, presumably by a strong wedging effect.

### H0 helix is necessary for the organization of the BAR assembly on a tube

**Figure 2:**
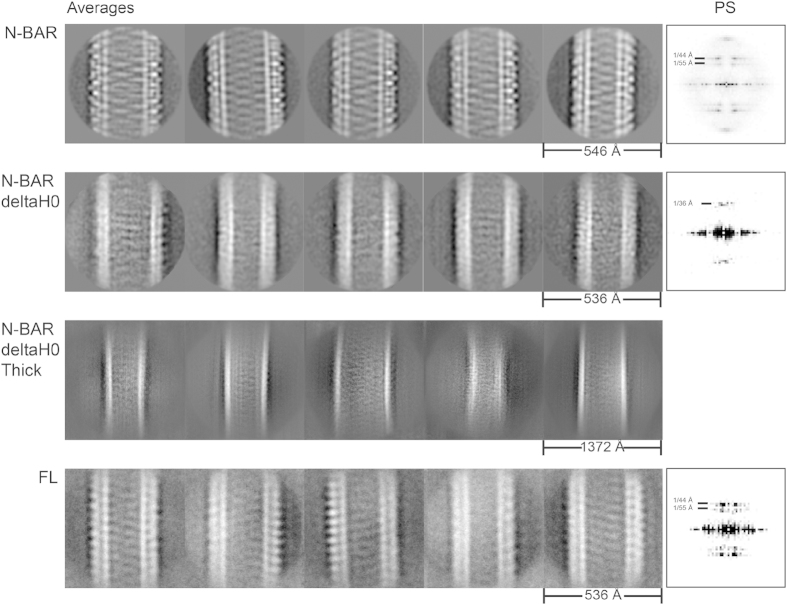
Left: Five representative 2D averages of tubes remodeled by N-BAR (first row), N-BAR-deltaH0 (second row), N-BAR-deltaH0 tubes with thicker size (third row), and FL (fourth row). For the N-BAR averages (first row, left to right), 1365, 1392, 1173, 692 and 1372 particles were used. The class averages of N-BAR-deltaH0 (second row, left to right) contain 234, 312, 165, 185 and 132 particles and 159, 403, 118, 282 and 447 particles for the thick class averages (third row, left to right). For FL (forth row, left to right) 398, 293, 284, 459 and 466 particles were used. Right: Power spectra of the most left 2D class averages. The 2D averages of N-BAR and FL reveal the organized protein assembly and the corresponding power spectra show periodical signal pattern of 44 and 55 Å corresponding to striped patterns within the tubes. N-BAR-deltaH0 reveals a spacing of ~36 Å and no interwoven pattern, indicating the protein assembly being not as rigid as in N-BAR mediated tubes and the change of the protein assembly.

To further assess the arrangement of the amphiphysin/BIN1 tube, remodeled tubes with amphiphysin/BIN1-FL, N-BAR and N-BAR-deltaH0 were observed using cryo-EM (Fig. 1D–1I). The majority of the tubes showed diameters of around 300 Å (Fig. 1J–L). Interestingly, when the N-BAR-deltaH0 is added, ~45% of tubes had a thicker morphology with a width of 650–850 Å (Fig. 1K). In these thick tubes, we did not identify any distinctive patterns (Fig. 1H, right) compared to the tubes with ~300 Å width (Fig. 1H, left). The 2D averages of the remodeled tubes generally exhibited striped patterns (Fig. 2), indicating proteins are making organized assemblies to some extent on a membrane surface. Particularly the 2D averages of the N-BAR-mediated tubes showed an interwoven pattern (Fig. 2, N-BAR “Averages”), which is an interfering moiré pattern of the overlapping the near and the far sides of the protein-coated tube as the cryo-EM image is a projection of a 3D object. The representative power spectrum revealed a typical diagonal pattern of periodical signals, indicative of a helical assembly of BAR proteins (Fig. 2, N-BAR, “PS”), agreeing with the assembly of other BAR protein-induced tubes^18^,^19^,^35^,^36^. The spacings of the protein assemblies derived from major layer lines are 44 and 55 Å (Fig. 2, N-BAR “PS”).

N-BAR-deltaH0 mediated tubes have patterns of BAR domains somewhat periodically arranged. However, the spacing was detected to be ~36 Å according to the power spectrum (Fig. 2, N-BAR-deltaH0, “PS”), in contrast to 44 or 55 Å of spacing observed for N-BARs. In addition we did not observe the interwoven pattern revealed from the N-BAR mediated tube. This indicates that the assembly of the N-BAR-deltaH0 is arranged enough to give a periodical pattern but it is not as rigid as the assembly of N-BAR. The change of the spacing indicates the H0 helix determines the arrangements of BAR assembly.

Together with the observation of tubular formation in a temporal manner, the role of amphiphysin H0 is likely to trigger the initial arrangement of the amphiphysin/BIN1, and to finally arrange an organized self-assembly of the BAR-units. However, H0 is not necessary for the amphiphysin/BIN1 mediated membrane tubulation itself.

### Regulatory domain is protruding out of the tube

It has been suggested that the ~8 kDa dynamin binding SH3 domain of the N-BAR protein endophilin is incorporated in the tube packing^19^. In the case of Drosophila amphiphysin/BIN1, the regulatory domain consists of 350 a.a., including a stretch of unknown function and a dynamin-binding SH3 domain. To assess the involvement of the regulatory domain of amphiphysin/BIN1 in the membrane remodeling activity, cryo-EM images of FL were evaluated. The cryo-EM images of the FL-induced tubes showed a similar tube formation as N-BAR with comparable width distributions (Fig. 1L). In addition, we observed needle-like densities protruding from the tubes (Fig. 1F,I, arrowhead). The 2D classification revealed an average with an interwoven lattice pattern (Fig. 2, FL “Averages”), although the average is not as defined as for the case of N-BAR, likely due to the overlap coverage of needle-like densities on the tubes. The power spectrum of the FL average showed the essential two periodical signals shown in the N-BAR 2D average (layer line, 44 and 55 Å^−1^), indicative of the same arrangement between FL and N-BAR mediated tubes. These observations indicate that the needle-like extra density is likely the regulatory domain and it is not incorporated in the tube lattice. It is rather likely that the N-BAR domain solely governs the tube formation.

Based on these morphological assessments, we have chosen to use the N-BAR protein fragment that contains BAR domains plus the N-terminal amphipathic helix (H0) as a minimal functional domain for observing further molecular interactions of amphiphysin/BIN1 in membrane remodeling.

### N-BAR makes a cooperative assembly on the membrane surface

In order to understand the effective protein concentration of amphiphysin/BIN1 for membrane remodeling, we tested the degree of tube formation as a function of protein concentration. Fluorescence light microscopy (Fig. S2A) as well as light-scattering measurements (Fig. S2B) showed that the critical concentration necessary for membrane remodeling in the presence of 720 μM liposomes is ~0.4 μM for the H0 containing protein fragments N-BAR and FL (Fig. S2B), while N-BAR-deltaH0 (Fig. S2) showed a critical concentration of ~1.6 μM. This indicates that H0 is needed for the efficient interaction of amphiphysin with membranes.

**Figure 3:**
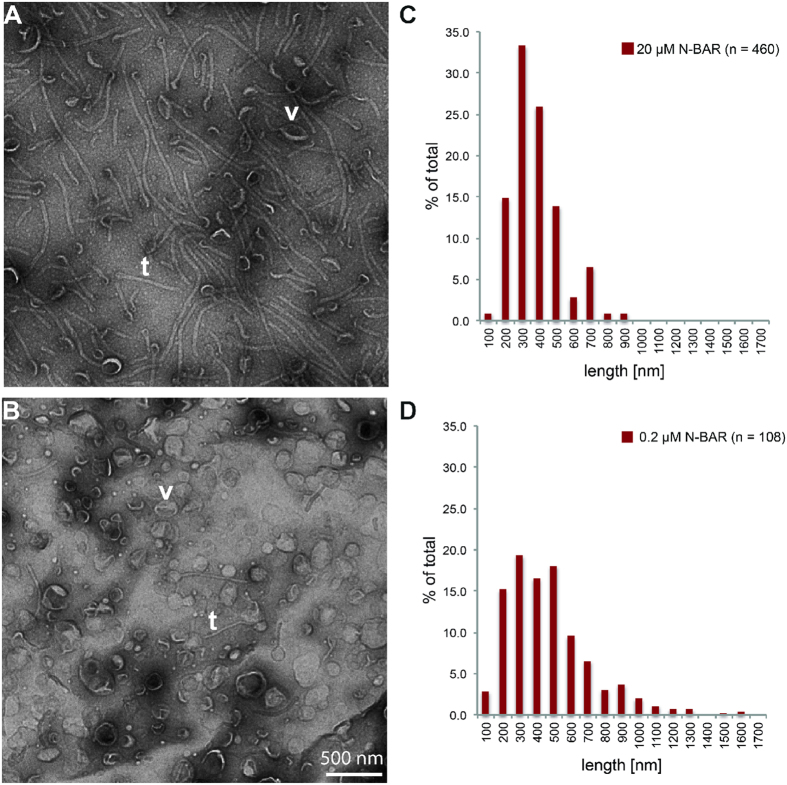
(**A**) Negative-stain EM observation of the N-BAR mediated tubes. 20 μM of N-BAR was added to 720 μM 200 nm-extruded liposome and after 10 min incubation, embedded on a grid without dilution. (**B**) 0.2 μM of N-BAR was added to the same liposome as (**A**). Most of the vesicles remained intact with this condition. v- examples of intact vesicles t: examples of tubes. (**C**) Histogram of the distribution of the lengths of the tubes shown in (**A**). (**D**) Histogram of the distribution of the lengths of the tubes shown in (**B**).

Further, negative-stain EM observations showed that most of the vesicles were remodeled into uniform tubes when 20 μM N-BAR was added to 720 μM 200 nm-extruded liposomes (Fig. 3A, t: an example of tube, v- an example of intact vesicle), and this condition was used for the cryo-EM based structural analysis (see below). As the protein concentration was lowered, a lesser degree of tubulation was observed and at around the critical concentration of 0.2 μM, vesicles are mostly intact with sparsely observed tubes (Fig. 3B). Strikingly, the tubes under these two conditions both have ~400 nm in length (see distributions of lengths, Fig. 3C,D). This means N-BAR assembly takes place in a cooperative fashion and suggests that the N-BAR has an ability of self-assembling on a lipid-membrane surface. If the protein assembly happens in a non-cooperative way, a larger number of shorter tube lengths or mild deformations of vesicles would be expected in the presence of the low concentration (0.2 μM) of N-BAR.

### N-BAR tubes show a packed assembly

Cryo-EM images of the amphiphysin/BIN1 N-BAR-coated tubes revealed a relatively uniform morphology of the amphiphysin/BIN1 assembly. Image analysis and classification yielded tubes with several distinctive widths ranging between 250–360 Å (Fig. 2, N-BAR, “Averages”, Fig. S3). Although the selected class averages showed distinctive features, we noted that the tubes were often bend, or the width of the tubes was not rigidly defined (Fig. 1D,G), which is in contrast to helical polymers of the cytoskeleton, yielding a robust structural analysis such as microtubules^38^ and actin^39^,^40^.

Nevertheless, to gain insight into the density of the N-BAR on the membrane tube, mass-per-length (MPL) analysis was performed using scanning transmission electron microscopy (STEM) (Fig. S4). The analysis of relatively straight tubes showed an average MPL of 28 ± 3 kDa/Å. The distribution of the MPL profile is wider compared to the rigidly organized TMV control (13 ± 0.7 kDa/Å), reflecting that the tubes are not as uniformly packed. Considering that the measured density is a sum of lipids and proteins, we estimated the contribution of the protein mass as follows. Lipid density is generally estimated as ~50 Å^2^/lipid^41^, therefore it can be estimated that ~18 lipids locate on a 300 Å-width tube per Å. This corresponds to ~14 kDa of mass. Therefore we estimated that the contribution of the mass of protein is ~14 kDa/Å. This led to the density estimation of one 56 kDa amphiphysin/BIN1 dimer to occupy 4 Å of axial space (along the tube axis), which agrees well with our 3D reconstruction (see below).

### 3D reconstruction shows a packed arrangement of N-BAR domains

**Figure 4:**
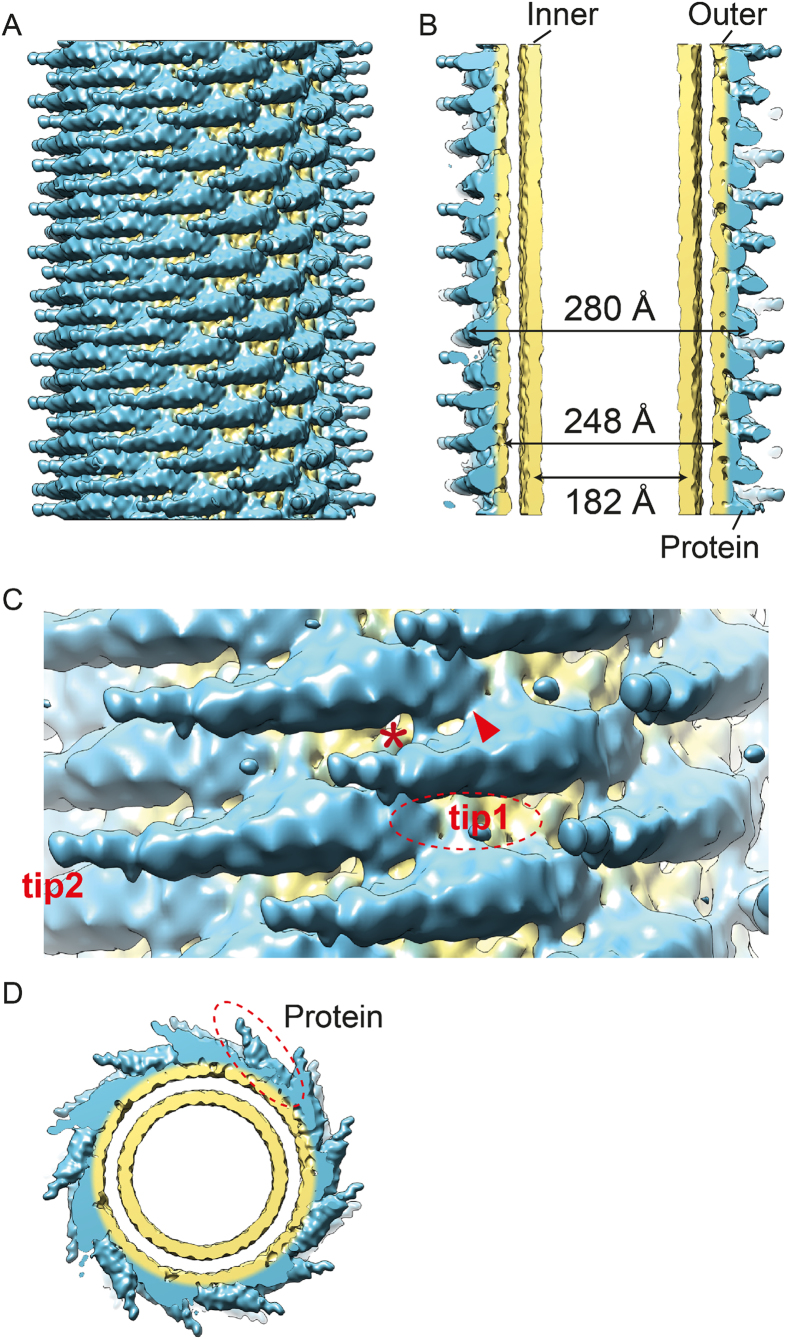
(**A**) 3D reconstruction of an amphiphysin/BIN1-mediated tube with a diameter of 280 Å. The density corresponding to the protein is colored in blue and the lipid corresponding parts are colored in in yellow. (**B**) The central portion of the 3D reconstruction is shown in (**A**). Inner leaflet (diameter of 182 Å) and outer leaflet (diameter of 248 Å) are colored in yellow. (**C**) Zoom in view of A. “*” indicates a rod-like density connecting adjacent BAR domains. The red arrowhead indicates an additional density connecting the adjacent BAR domains. “Tip1” shows a tip density hidden under the membrane. “Tip2” shows the second tip of the BAR unit. (**D**) End-on view of (**A**).

To see the N-BAR assembly on a tube, we chose 5 classes with distinctive features for further image analysis (Fig. 2, first row and S5, column “Averages”). The 3D reconstructions of particles from these classes revealed protomers of N-BAR dimers wrapping around the tubulated liposomes (Fig. 4A,B, Fig. S6). Reflecting its observed flexibility in packing, the resolution of the reconstruction appeared limited to a medium range resolution of ~11 Å (Fig. S5, column “FSC” and methods). The arrangement of the protomers showed a tightly packed organization of the BAR assembly. The helical rise was ~ 3.8 Å (see methods), well agreeing with 4 Å/BAR unit from the STEM measurement and corresponding to a translation of the BAR protomer of a 1-start helix towards the axis of the tube. The line-scan of the reprojection of the 3D reconstruction across the tube axis showed three peaks from the center of the tubes, corresponding to the inner leaflet, outer leaflet of the lipid bilayers and the attached protein density (Fig. S5, column “model radius”). The distance between inner and outer leaflets of the tube bilayer was measured to be ~33 Å.

The area occupied by one N-BAR unit (i.e. dimer) is 3000–4000 Å^2^, varying depending on the diameter of the tubes, while the concave surface area of amphiphysin is calculated to be ~10,000 Å^2^. This is in contrast to ~18,000 Å^2^ calculated from the loosely packed version of endophilin BAR protein-remodeled tube^19^. The tightly packed arrangement is achieved by one tip of the BAR unit hidden underneath the membrane surface (Fig. 4C, tip1), while the other tip protrudes out from the surface of the tube (Fig. 4C, tip2). Due to this tight packing of the BAR units, the tip-to-tip arrangement observed for endophilin^18^ or the CIP4 F-BAR domain^35^ was not seen in the case of amphiphysin/BIN1. Rather, the interaction between neighboring BAR units was detected between the central portion of one BAR unit and a protruding tip of the neighboring BAR unit (Fig. 4C, marked as *). The BAR domain is attached on the outer membrane leaflet (Fig. 4B,D, Fig. S5, column “model radius”). The tip of the BAR domain protruding out from the surface of the membrane was responsible for the jaggy features on the side of the tubes seen in 2D averages (Fig. S5, column “Averages”) and in the re-projection of the 3D reconstruction (Fig. S5, column “Reprojections”).

We detected several classes of tubes with various diameters (Fig. S5, column “model radius”, Fig. S6). The major class with the most distinctive features (1948 segments) has a diameter of 280 Å (Fig. 4). There was one class with a wider diameter (312 Å), identified discretely (Fig. S6A). In this class, a slightly less tight packing of the BAR units was revealed. In contrast, we detected populations of tubes whose diameters were smaller (240–260 Å), but appeared transiently fluctuating (Fig. S5, third to fifth row, and Fig. S6B–S6D).

### Fitting of an atomic N-BAR model shows that multiple interfaces of the BAR domain can be involved in the tube assembly.

**Figure 5:**
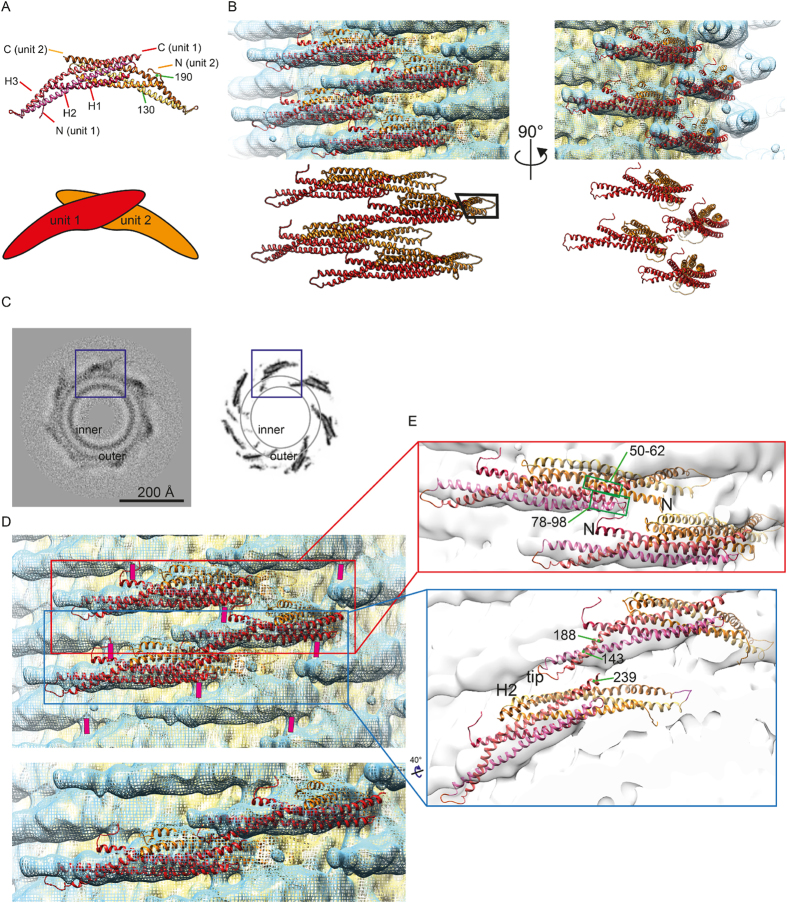
(**A**) Atomic model (PDB code 1URU) of Drosophila amphiphysin (top). The crescent BAR unit is achieved by dimerization (unit 1 and unit 2, bottom). The amphipathic H0 helix is not visualized, as it is unstructured in the crystallization condition. (**B**) Fitting of the atomic model shown in (**A**) to the amphiphysin mediated tube of 280 Å –diameter. The black box highlights the tip portion not visualized by the reconstruction. (**C**) The density of a section of the 3D reconstruction (left) in comparison to the densities of the amphiphysin atomic model fitted and symmetrized according to the arrangement calculated from the 3D reconstruction. Inner/outer: headgroup density of the inner or outer leaflet. One tip of the BAR is immersed into the acyl chain region according to the rigid body fitting. This part of the reconstruction was not resolved due to the surrounding lipids (boxed in (**B**)). (**D**) (Top) The rod-like density connecting the BAR units are marked with a pink bar. (Bottom) A cropped representation of (top) without the pink marker. (**E**) Representation of the atomic models showing the arrangement of the neighboring BAR units.

The fitting of the crystal structure (PDB code 1uru) to the 3D reconstructions revealed the molecular organization of the BAR domains necessary to achieve the lattice packing of amphiphysin/BIN1-mediated tubes. The amphiphysin/BIN1 dimer consists of three alpha helices forming an anti-parallel coiled-coil in each monomer, resulting in a six-helix bundle (Fig. 5A).

The molecular fitting of the atomic model to the reconstruction from the major class with 280 Å diameter (Fig. 5B) showed that almost one fourth of the BAR dimer unit had a direct connection to the membrane surface (Fig. 5B, boxed area). This area includes approximately residues 130–190 in helix 2 and helix 3 (Fig. 5A,C) and a loop connecting these two helices. In particular, the tip area appeared immersed and surrounded by lipids possibly up to 9 Å in depth (Fig. 5C). This observation was in agreement with the recent EPR results showing residues 144, 147 and 151 to be deeply immersed into the acyl chain region of the lipids^13^. The tip region of the atomic model did not precisely fit to the tested EM map (Fig. 5D). This observation was consistent among all calculated reconstructions of various classes. This suggests that there could be a local geometrical rearrangement or fluctuation of the tip region upon membrane interaction and the lattice formation.

The inter-dimer connections between neighboring BAR units are visible (Figs 4C * and 5D, guided with pink bars). These densities are not occupied by the amphiphysin/BIN1 crystal structure. This is likely the H0 helix, which is not included in the crystal structure due to its unstructured nature without membrane. This connection is made around the junction between H1 and H2 (around residues 78–98) of a monomer and/or H1 (around residues 50–62) of the paired monomer within the dimer unit (Fig. 5E). The second H0 helix in the BAR dimer unit was not resolved. The immersion of the tip density into the membrane portion has hampered the visualization of the H0 helix, although there is a small connection appearing to be a candidate (Fig. 4C, marked with red arrowhead).

The reconstruction with a wider diameter (312 Å, Fig. S6A) showed that the BAR domain was arranged slightly differently. The area occupied by one BAR unit increased to 3700 Å^2^, compared to 3300 Å^2^ in the class shown in Fig. 4. The interaction points are shifted by ~25 Å away from each other (Fig. S6A) compared to the reconstruction of 280 Å in diameter (Figs 4 and 5). In contrast, with narrower tubes, the crescent shaped BAR protein appeared to rotate along its long axis (Fig. S6, B–D). The dimer-dimer interaction surfaces were changed by the rotation of the BAR rather than a translation. We did not observe any obvious additional connections between adjacent BAR units in either of these reconstructions.

### BAR tip is not necessary for tubulation but required for organized arrangement for the N-BAR lattice formation

All of the reconstructions showed that one tip of the BAR unit was protruding out from the tube, while the other tip was immersed into the membrane. To test the effect of the tips of the BAR units in tubulation, we made a mutant that lacks the tip portion (residues 147-176) (N-BAR-delta-tip) and an obligated amphiphysin/BIN1 N-BAR fusion heterodimer that contains an intact N-BAR domain of amphiphysin/BIN-1 and an N-BAR domain that lacks the tip portion (N-BAR-N-BAR-delta-tip) and assessed its membrane-remodeling ability. Interestingly, we observed that these protein fragments were also able to remodel a membrane surface to a tube (Fig. S7). The critical concentration of the N-BAR-delta-tip was measured to be 0.7 μM, slightly higher than the case for N-BAR (0.4 μM). The N-BAR-N-BAR-delta-tip showed a critical concentration of 0.2 μM, the lowest compared to all tested protein fragments. However, we observed a sporadic population of bundling of the tubes, possibly due to a re-arrangement of some of the BAR domains and the formation of inter-dimer, i.e. inter-tube crosslinks. This inter-dimer formation may increase the local protein concentration, thus, leading to a decrease in the critical concentration.

The negative-stain EM showed that these tubes are similar in size to the tubes mediated by wild-type fragments, but the internal order of the tubes was completely lost. This suggests that both tips are necessary for the organized membrane remodeling but not required for the membrane curving activity.

## Discussion

### Tight packing of amphiphysin/BIN1-mediated tube assembly and the structural scaffolding function

**Figure 6:**
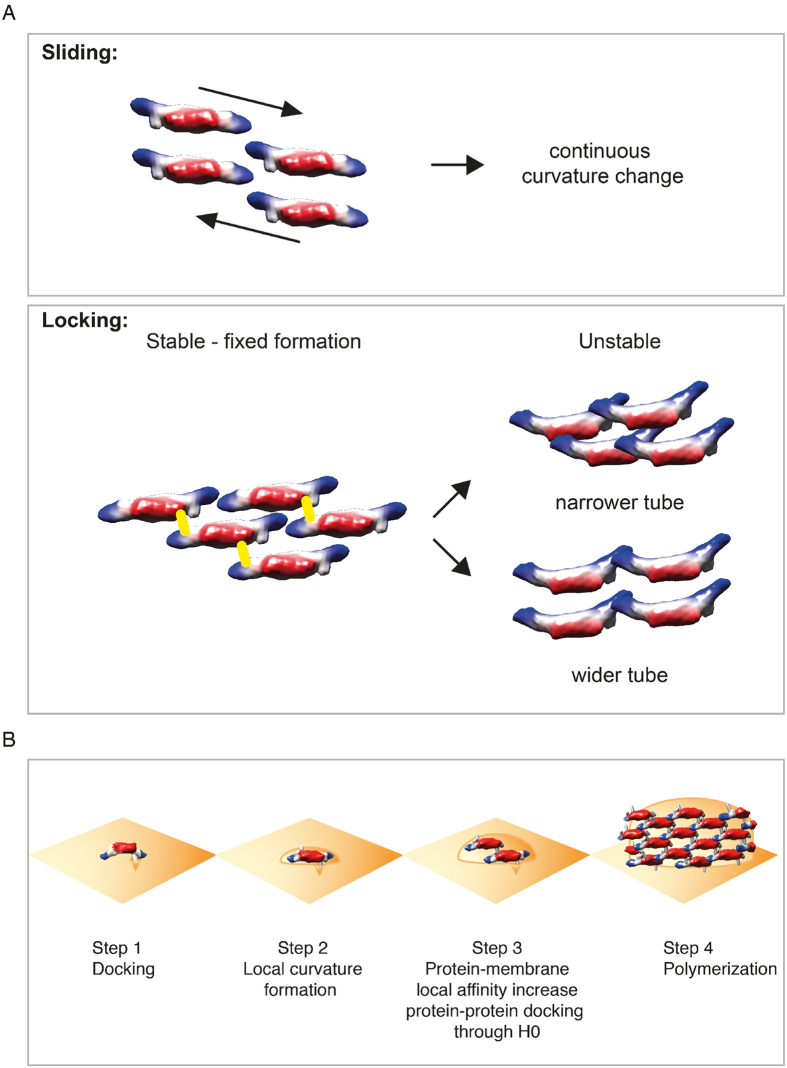
(**A**) A model how different BAR proteins are achieving membrane remodeling. (Top): Sliding mechanism, showing that neighboring BAR units have weak interactions with each other so that they may slide against each other to achieve a continuously changing curvature. This example is shown in endophilin-mediated tube formations^18^. (Bottom): Locking mechanism, showing that the BAR units have a preferred rigid formation with a help of H0, however, the BAR units can rotate along their long axis, therefore losing the connection point via the H0 helix and resulting in a variation of the diameter, i.e. curvature. When the balance of the tube arrangement is too disturbed, N-BAR proteins may even squeeze the tubes to produce small vesicles. (**B**) A model of the cooperative assembly of the N-BAR proteins depicting the wedging effect of the amphipathic H0 helix and the BAR scaffold. In this model, a change of the local curvature of the membrane caused by the landing of the first N-BAR unit is the driving force of the N-BAR cooperative assembly.

The 3D reconstructions of amphiphysin/BIN1-mediated tubes showed an intriguing difference from tubular formations facilitated by other BAR protein assemblies^18^,^19^,^36^. Particularly, endophilin and amphiphysin/BIN1 have very similar crystal structures^10^,^11^,^42^,^43^ but we showed considerable differences in the packing in the presence of membranes. We showed that amphiphysin/BIN1-mediated tubes have a significantly higher degree of rigidity. This may be due to differences in the packing of the BAR units. The protein packing is much tighter in amphiphysin/BIN1 compared to endophilin-mediated tubes^18^,^19^ and the neighboring BAR units of amphiphysin/BIN1 are well connected with each other. This may reflect the physiological role of amphiphysin/BIN1’s function in giving a stable structure in muscle T-tubules, while endophilin is rather involved in a dynamic membrane curving process during endocytosis. The membrane squeezing process occurs through the inter-dimer connection. We have previously proposed a sliding mechanism for the case of endophilin, i.e. the interaction of the inter-dimer surface may not be based on specific electrostatic interactions but rather on shallow electrostatic surface potentials and therefore a continuous change of curvature is achieved (Fig. 6A, top). With amphiphysin/BIN1, we detected a population of a few tubes with relatively distinctive sizes, and the most rigidly arranged tube revealed a density linking a perpendicular connection between parallel arranged neighboring BAR domains. This connecting density is likely presenting the H0 helix, as this rod-like density is located proximal to the N-terminus of the BAR domain. Further, we did not detect such a rod-like density in the narrower tubes where BAR units appeared to be rotated along its long axis and which showed fluctuations in tube size. It appears that amphiphysin/BIN1 locks the interactions between the neighboring BAR domains in a discrete fashion and this locking is reinforced by H0 (Fig. 6A, bottom). When the interactions of H0 are lost and the fluctuation of the protein goes beyond a certain balance, the squeezing force may pinch off the tube to create small vesicles. The local fluctuation in the packing hampers a high-resolution structural analysis and it may be an intrinsic property of BAR protein-based assemblies on a flexible membrane platform.

### Role of amphiphysin/BIN1 in T-tubule biogenesis

During the T-tubule development, it is thought that amphiphysin/BIN1 is responsible for the remodeling of the membrane into a tube^25^. Caveolin 3 is proposed to be involved in the early stage of T-tubule biogenesis^44^ by forming caveolae at the plasma membrane and amphiphysin/BIN1 invaginates the plasma membrane deeper. Although co-localization of caveolin 3 and amphiphysin/BIN1 is observed^25^, no direct interaction between both proteins has been reported so far and the recruitment process of amphiphysin/BIN1 is unclear. On the other hand, it is reported that amphiphysin/BIN1 preferably binds to PtdlIns(4,5)P2 through its BAR domain and clusters it. Subsequently, amphiphysin/BIN1 recruits the downstream player dynamin with its SH3 domain^45^. Therefore, it seems that PtdIns(4,5)P2 is key for the recruitment of all involved players. From this point of view, it is plausible that the local concentration of PtdIns(4,5)P2 may already take place during the caveolae formation for the efficient recruitment of amphiphysin/BIN1. The cooperative assembly of amphiphysin/BIN1 on a membrane surface may also play an important role for triggering the local clustering of necessary components and enrichment of PtdIns(4,5)P2. The biochemical and structural studies of these molecular interactions are important topics of further research.

### Influence of the CNM causing mutation to the BAR-assembly

The mutations that cause the disease CNM are located at H0 (K35N, corresponding to K30 in Drosophila amphiphysin/BIN1), D151N (corresponding to D146) and R154Q (corresponding to R149) at the tip of the crescent BAR unit. This is consistent with our results that H0 and the tip areas are critical for the organized protein assembly. It also agrees well with previous *in vitro* studies^13^,^46^. Particularly, a recent report by Isas *et al.*^13^ showed biophysically that the tip of the BAR unit including D151 is deeply immerged into the membrane, up to a hydrophobic lipid acyl chain region and the authors discussed the importance of this observation in the context of tubulation. This finding agrees well with our direct observation that the tip of one BAR unit is inserted in the lipid bilayer, although the other tip appears to be protruding out from the membrane surface. Interestingly, we noted that the deletion mutant that misses significant parts of the tip is still able to remodel membranes. The negative-stain EM imaging of the resulting tubes did not reveal any representative pattern as seen in the N-BAR remodeled tubes, suggesting a lack of arranged organization of the BAR domains (Fig. S7). This was also true for the fusion protein fragments that have one tip missing. Likely, one tip of a crescent BAR domain is beneficial for membrane tubulation by directly interacting with lipids, while the other tip is important for the lattice formation and stabilization by interacting with the adjacent unit (Fig. 5). We observed a similar effect with the BAR fragment missing H0. Without H0, the organization of the BAR domain is partially lost likely by missing the density that connects adjacent BAR units (Fig. 5D, pink bar), even resulting in the shrinkage of the packing indicated by the power spectrum (Fig. 2). The defects of the tip mutation or deletion are subtle in terms of tubulation activity but they are disrupting the ultrastructure of the amphiphysin/BIN1 self-assembly, which likely connects to the disorganization of the T-tubules. We note that we have employed a specific lipid composition to facilitate structural analysis^13^, but differences may be more pronounced under physiological lipid conditions.

### Role of the H0 helix for the initiation of membrane remodeling

In our study, we hinted the role of the wedging by the H0 amphipathic helix and the scaffolding by the BAR crescent-like structure. For membrane remodeling itself to take place, H0 is not necessary. However, H0 is required to achieve an efficient and regular arrayed assembly of the BAR polymers. From these observations, we surmise that the amphipathic helices may be necessary for the initiation of curvature induction and the arrangement of BAR dimers in an organized fashion – poised to polymerize (Fig. 6B). The initial membrane induction from a flat to a curved membrane surface may occur through its wedging function of H0 (Fig. 6B, step1 and 2). When the membrane curvature fits to the crescent shape of the BAR domain, the BAR scaffold may efficiently attach and fix the membrane curvature. This process is more effective at the proximal vicinity to a membrane surface where the first BAR domain has already landed and pre-fixed the local membrane curvature (Fig. 6B, step3). The proteins may start arranging guided by this given membrane curvature and the final stabilization of the protein polymer may be held through the H0 connections (Fig. 6B, step4). This notion agrees with the previous observation that the local concentration of amphiphysin correlates with the curvature of membranes given^47^. Without H0, the initial protein binding to the membrane may be achieved by simply sensing the membrane curvature that is stochastically fluctuating. Hence, this process may be too slow for physiological requirements. On the other hand, the tip portion of the BAR may not be as important in terms of initial curvature formation as H0, as the critical concentration necessary for membrane remodeling for the N-BAR-delta-tip (0.7 μM) did not increase as much as the case for the N-BAR-deltaH0 fragments (1.6 μM).

## Methods

### Cloning and protein expression

The amphiphysin/BIN1 gene was obtained from the Drosophila cDNA library, the Berkeley Drosophila Genome Project (BDGP) Gold cDNA Collection (Drosophila Genomic Resource Center). The gene was amplified by PCR and cloned into self-generated pEC series vectors designed for Ligase Independent Cloning^48^. Recombinant amphiphysin/BIN1 N-BAR (aa 1-244 or aa 1-247), N-BAR-deltaH0 (aa 27-247 or 31-244), deltaH0 (aa 27-602 or 31-602) and FL (1-602) were cloned as 3C-protease cleavable hexahistidine (His) or SenP2-protease cleavable sumo-hexahistidine fusion proteins. For N-BAR-delta-tip protein fragments, residues 147-176, corresponding to the tip part of the BAR were deleted and the residue 146 and 176 were connected by (GS)5 linker by PCR. The N-BAR-single-tip-fusion dimer (N-BAR-N-BAR-delta-tip) was constructed by amplifying N-BAR and N-BAR-delta-tip with a (GS)7 or (GS)8 linker by PCR, respectively, and fusing them by ligation-independent cloning. In brief, T4 polymerase was used to generate complementary overhangs on both inserts that could be readily annealed with each other and the target vector. The fusion proteins were expressed in *E.coli* BL21 gold using auto-induction ZY media^49^ supplemented with appropriate antibiotics. The bacterial cultures were grown at 37 °C until they reach an optical density of around 2. Afterwards the temperature was reduced to 18 °C and proteins were expressed over night. The cells were harvested by centrifugation (8000 × g, 10 min) and stored at −80 °C until further usage.

### Protein purification

The harvested cells of recombinant amphiphysin/BIN1 fragments were lysed in 20 mM Hepes, pH 7.4, 500 mM NaCl, 1 mM DTT supplemented with 1 mM Pepstatin A, 1 mM AEBSF and 1 mM leupeptin. The soluble fraction was loaded on a His-Trap column (GE Healthcare). The column was washed with 20 mM Hepes pH 7.4, 500 mM NaCl, 250mM imidazole, 30 mM imidazole, 1 mM DTT. The protein was eluted with 20 mM Hepes pH 7.4, 300 mM NaCl and 1 mM DTT. The sumo-His-tag was cleaved by SenP2-protease over night at 4 °C. Afterwards for N-BAR and N-BAR-deltaH0, N-BAR-delta-tip, and N-BAR-N-BAR-delta-tip, a cation exchange chromatography and for deltaH0 and FL an anion exchange chromatography was carried out (gradient of Buffer A: 20 mM Hepes ph 7.4, 1 mM DTT and Buffer A2: 20 mM Hepes pH 7.4, 2 M NaCl, 1 mM DTT). For further protein purification, size exclusion chromatography (SEC) was followed using a Superdex 200 16/60 column (GE Healthcare) with 20 mM Hepes pH 7.4, 500 mM NaCl, 1 mM EDTA and 1mM DTT. The purified proteins were stored at −80 °C until further usage.

### Liposome preparation and *in vitro* tubulation assay

The following synthetic lipids were used: POPG, POPE and POPC (Avanti Polar lipids). Additionally a bovine brain extract, Folch Fraction I (Sigma Aldrich) was used. For tubulation experiments large uni-lamellar vesicles (LUVs) or multi-lamellar vesicles (MLVs) were prepared. The dried lipid film, containing 2POPG:1POPE (w/w) was hydrated with buffer (20 mM Hepes pH 7.4) to achieve MLVs. LUVs were obtained by extrusion with a filter membrane (200 nm) (Avanti Polar lipids). The lipid condition was determined based on the screenings done by Isas *et al.*^13^. All prepared lipids were either immediately used or stored at 4 °C for 2–3 days.

For the tubulation kinetics experiments various amphiphysin/BIN1 fragments (20 μM, 15 μM or 6 uM of N-BAR and N-BAR-deltaH0 or 6 μM of FL and deltaH0) were incubated with 180 μM of liposomes. The tubulation was measured by absorbance spectrometry at 400 nm and by negative-stain EM. For tubulation assays the absorbance was followed for 120 min. Additionally, aliquots of different proteins were taken at several time points up to 45 min and samples were assessed with negative-stain EM.

### Tube length, diameter and critical tubulation concentration determination

20 or 0.2 μM of N-BAR was added to 720 μM 200 nm-extruded LUVs and after 10 min incubation, embedded on a grid without dilution and stained with 1% (w/v) uranyl-acetate staining dye and screened by CM200 (FEI) operating at 160 kV. The images were recorded by CCD camera with the nominal magnification of 50,000× corresponding to 2.16 Å/pixel.. The N-BAR tube length was determined from negative-stain EM images with the “filament” option in bshow (bsoft package)^50^. Afterwards the measured values were evaluated by a histogram. The tube diameter was determined and calculated by plotting the class averages from cryo-EM dataset or from the reconstruction reprojections. The distributions of the tube diameters were analyzed by histogram. This was done for N-BAR, N-BAR-deltaH0 and FL. The critical tubulation concentration was determined by fluorescence light microscopy and light scattering measurement. For the fluorescence light microscopy 1% Atto488-DOPE was added to the liposomes (w/w 2POPG:1POPE) and after the preparation 720 μM of liposomes were used with various dilutions (6, 0.6, 0.06, 0.006 μM) of N-BAR, N-BAR-deltaH0, FL, N-BAR-delta-tip, N-BAR-N-BAR-delta-tip for observing the degree of tubulation. For the image recording a GE Deltavision Elite with a 60x/oil or 100x/oil objective and excitation 475 nm/emission 525 nm bandpass filter was used. Light scattering was measured to quantify the critical tubulation concentration. Various concentrations of of N-BAR, N-BAR-deltaH0, FL, N-BAR-delta-tip, N-BAR-N-BAR-delta-tip were mixed with 720 μM 2POPG:1POPE (w/w) and the absorbance change was observed. Due to the saturation of the signals at 400 nm, the measurements were performed at 490 nm wavelength.

### STEM measurement

The N-BAR induced tubes were prepared in 20 mM Hepes, pH 7.4. For the STEM dark-field imaging, the samples were prepared in the standard procedure of the Brookhaven STEM facility. On the glow discharged carbon grids 3–5 μl of freshly mixed 20 μM of N-BAR and 720 μM of liposomes was added and incubated for 1 min. Then the specimen was washed for few times and afterwards plunged into liquid nitrogen for fast freezing. The grid was freeze-dried overnight and transferred under vacuum into the STEM. For the data analysis dark-field digital micrographs with a pixel size of 1 to 2 nm/pixel were used and analyzed by PCMass32 (available from the Brookhaven STEM resource, ftp.stem.bnl.gov). As an internal mass per length reference tobacco mosaic virus (TMV) (13.1 kDa/Å) was included in all samples. The N-BAR or TMV were boxed according to their size and through this the mass per length was measured. A histogram was plotted from these data.

### EM grid preparation and image acquisition for negative stain and cryo-EM

EM observations were performed either with 20 μM proteins and 720 μM liposome, or 5 μM proteins and 180 μM liposome (4 times dilution). The negative staining samples were stained with 1% (w/v) uranyl-acetate staining dye and screened by CM200 (FEI) operating at 160 kV. The images were recorded by CCD camera with the nominal magnification of 50,000× corresponding to 2.16 Å/pixel.

The cryo-EM samples were applied to glow-discharged Quantifoil grids (Cu, R 2/1), incubated for 10 s, then manually blotted for 10 s with filter paper (Whatman No. 1) and vitrified with ethane by a manual plunger. The cryo-EM specimens were observed using a Tecnai F20 microscope (FEI) operating at 120 kV and 200 kV with a magnification of either 62,000× or 80,000 ×, resulting in a pixel size of 1.78 Å/pixel and 1.34 Å/pixel, respectively, and recorded with a CCD camera (FEI, Eagle) by using Serial EM software. The defocus range was 1–3 μm. Additionally, cryo-EM data sets were also taken with a Polara G2 F30 microscope (FEI) operating at 300 kV with a magnification of 62,000 × and recorded with a Gatan K2 summit direct detector (3838 × 3710 pixel) and a GIF 2000 energy filter, corresponding to 1.82 Å/pixel using Serial EM software. In every grid hole only one position was imaged with a total exposure of 5s and a frame time of 0.1 s. For the image processing frame 10 to 40 was used with a total electron dose of 30 electrons per Å^2^. The defocus range for of the data sets was 0.5–3.2 μm.

### Image processing of cryo-EM data set

For the image processing the frames were aligned and the drifting of frames was corrected. The tubes were segmented by helixboxer of EMAN2^51^. 26754 segments were extracted with a box size of 300 pixels with an overlap of 90%. The defocus and astigmatism were determined by ctffind3^52^ and afterwards the contrast transfer function was corrected by phase flipping by bctf from BSOFT software package^50^. All segments were 2D classified by Relion^53^. During this process 19423 segments were discarded due to bad quality. The result of the final round of the 2D classification is shown in Fig. S3.

We chose 5 classes for further assessment. These 5 classes have diameters of 280, 312, 262, 250, 242 Å and 1948, 1392, 692, 1173, 1372 segments respectively.

The reconstructions using particles from individual classes were performed by IHRSR^54^ implemented into SPIDER software package^55^. As an initial model, a Gaussian noise filled cylinder with the same diameter as the test data was used. The first rounds of the reconstruction were performed using 2x binned images with an azimuthal increment of 4 degree, without the search for out-of-plane tilt, to accelerate the process. During the refinement, the azimuthal increment was set to 1 degree with an out-of-plane tilt of 1 degree increment up to +/– 10 degree, thus the references of 7200 reprjection images were created for projection matching. “hsearch” in IHRSR software package^54^ was used to find a local conversing point, but this option was not used for initially determining the helical parameter. Instead, the initial helical parameters of individual class were calculated from the 2D class average and the corresponding Fourier Transform. Considering some heterogeneity, we calculated several possibilities and assessed the resulting 3D reconstruction by comparing the reprojections and the class averages and their power spectra. After the processing, the final helical parameters converged to a rise per subunit of 3.81 Å, an azimuthal rotation of 55.92° for the class shown in Fig. 4 and Fig. S5, first row. The other classes showed similar helical parameters (∆z, ∆phi) as 3.83 Å, 55.96° (Fig. S5, second row, Fig. S6A), 3.84 Å, 56.01° (Fig. S5, third row, Fig. S6B), 3.82 Å, 55.93° (Fig. S5, fourth row, Fig. Fig. S6C) and 3.85 Å, 55.99° (Fig. S5, fifth row, Fig. S6D).

The resolution of all reconstructions was determined by the Fourier shell correlation (FSC) of the half data sets at the cut off 0.5. To calculate the resolution, we performed a few cycle of the IHRSR reconstructions to assure the independence of the reconstructions. The resolutions were calculated to be 10.3, 11.2, 10.9, 10.9, 12.1.

### Fitting of the amphiphysin BAR crystal structure

The known crystal structure of Drosophila-amphiphysin BAR (PDB code 1URU) was manually fit into the reconstructed density of a BAR dimer. In order to perform amplitude correction of the 3D reconstructions, the fitted BAR dimer atomic model was converted to a density map using Chimera software and the helical symmetry of the reconstruction was imposed by bhelix (bsoft package). This helically symmetrized BAR map was used as a reference for the amplitude correction of the reconstruction. Bampweigh (bsoft package) was used for the amplitude correction. The final maps were low-pass filtered to 11–12 Å. BAR dimer interactions were analyzed using the program Chimera.

## Additional Information

**Accession codes**: The reconstruction files are deposited in the Electron Microscopy Data Bank (EMDB) under the following accession number: 3192, 3193, 3194, 3195, 3196.

**How to cite this article**: Adam, J. *et al.* Structural insights into the cooperative remodeling of membranes by amphiphysin/BIN1. *Sci. Rep.* **5**, 15452; doi: 10.1038/srep15452 (2015).

## Supplementary Material

Supplementary Information
