# Supplementary material for: Structural insights into the cooperative remodeling of membranes by amphiphysin/BIN1
Source: Sci Rep. 2015 Oct 21;5:15452. doi: 10.1038/srep15452 (PMC4614383; doi:10.1038/srep15452)

Structural insights into the cooperative remodeling of membranes by amphiphysin/BIN1

Julia Adam, Nirakar Basnet and Naoko Mizuno*

Cellular and Membrane Trafficking,
Max Planck Institute of Biochemistry, Am Klopferspitz 18, D-82152 Martinsried, Germany

*Corresponding author
E-mail: mizuno@biochem.mpg.de
Phone: + 49 (89) 8578 – 3479
Fax: +49(89) 8578 – 3605

Supplementary Figure Legends

Figure S1

Tubulation of various amphiphysin fragments with vesicles observed by absorbance measurement and negative staining. (A) Absorbance measurement at 400 nm of 180 μM vesicles mixed with 5μM N-BAR, FL, N-BAR-deltaH0 and deltaH0. The increase in the absorbance at 400 nm corresponds to tube formation. The negative control shows the absorbance of vesicles without any protein. (B and C) Negative stain EM observation of (B) vesicles and (C) tube formations of NBAR-deltaH0 (left) and N-BAR (right). The tubes are observed immediately after mixing (22 sec) and at various time points (2 min, 10 min, 30 min and 45 min) up to 45 min. After ~30 min of incubation, N-BAR-mediated tubes visibly transform into small vesicles (right, bottom, red arrow heads).

Supplementary Fig. S1

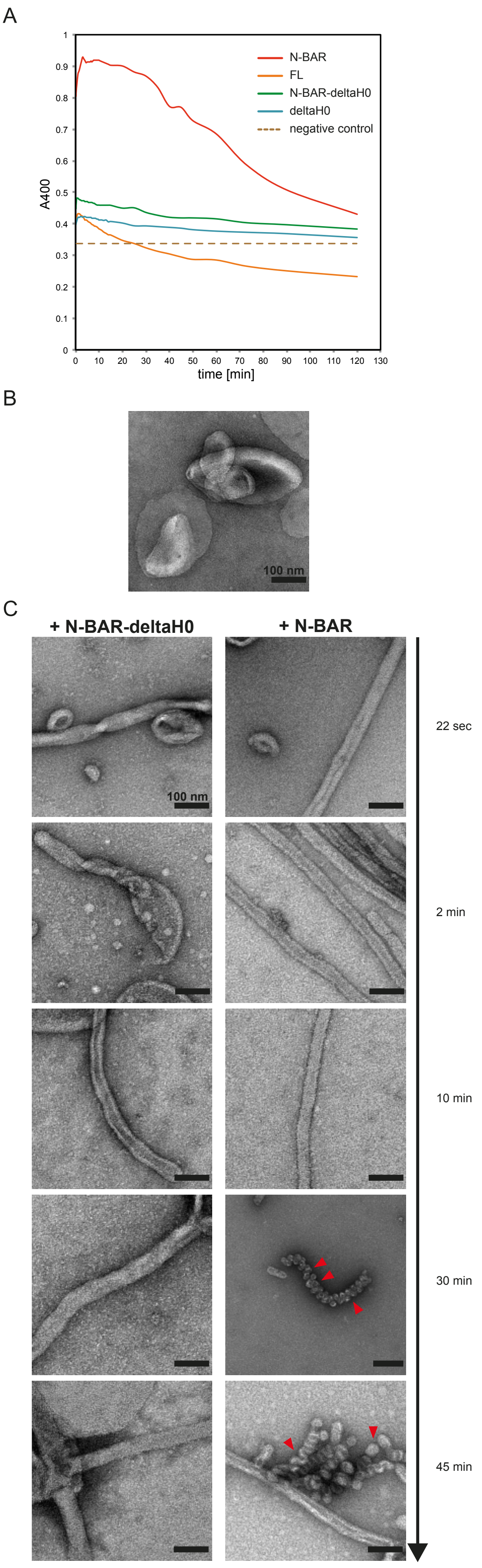

Figure S2

(A) Tubulation observed by fluorescence light microscopy. Various protein dilutions (6 µM, 0.6 µM, 0.06 µM, 0.006 µM) of N-BAR, N-BAR-deltaH0 and FL were incubated with 720 µM of fluorescently labeled vesicles. N-BAR and FL show distinct tubulation up to a concentration of 0.6 µM. In contrast N-BAR-deltaH0 mediated tabulation is only observed with 6 µM of protein. The degree of tubulation is described as ++ > + > +/- (tubulation very sparsely happening) > - (no tubulation). v: examples of vesicles and t: examples of tubes. (B) The light scattering of the mixture of 720 µM vesicles and various concentrations of N-BAR (left), N-BAR-deltaH0 (middle) and FL (right). The graphs show the critical concentrations where no tubulation occurs anymore (guided with flat lines) of 0.4 µM (N-BAR and FL) and 1.6 µM (N-BAR-deltaH0).

Supplementary Fig. S2

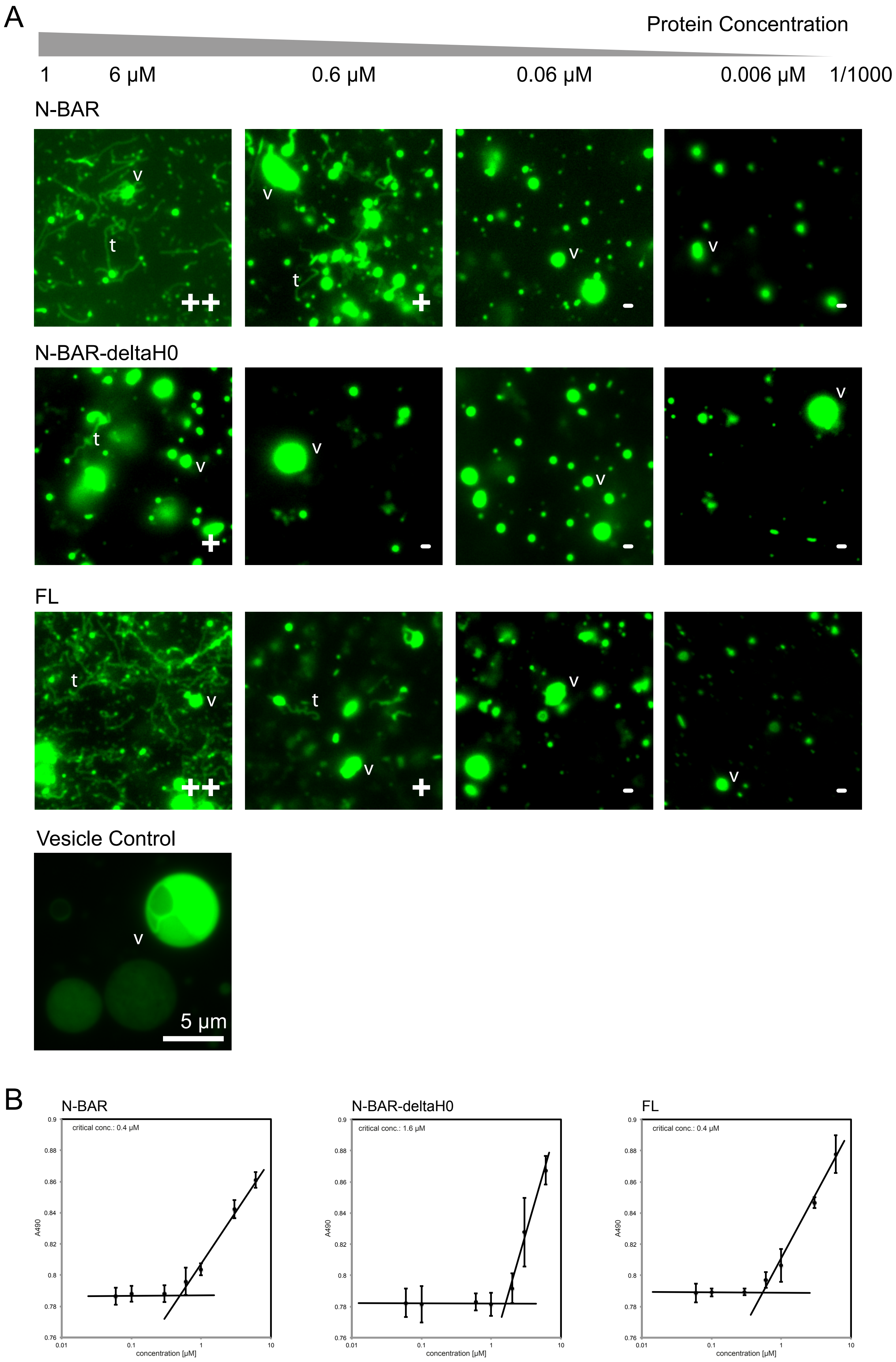

Figure S3

2D averages of N-BAR mediated tubes. After CTF correction (phase-flipping) of the collected micrographs, the N-BAR mediated tubes were boxed into 26754 segments (300x300 pixel, corresponding to 546 x 546 Å). The segmented tubes were classified with reference free classification scheme provided by Relion software. To achieve 2D averages with better features, bad segments were removed by several classification iterations. After removal of bad segments around 19423 boxed segments were used to obtain 50 final 2D class averages. The particles in the classes with the most distinctive features were used for further data analyses and 3D reconstructions as shown in Figure S5.

Supplementary Fig. S3

N-BAR tube class averages

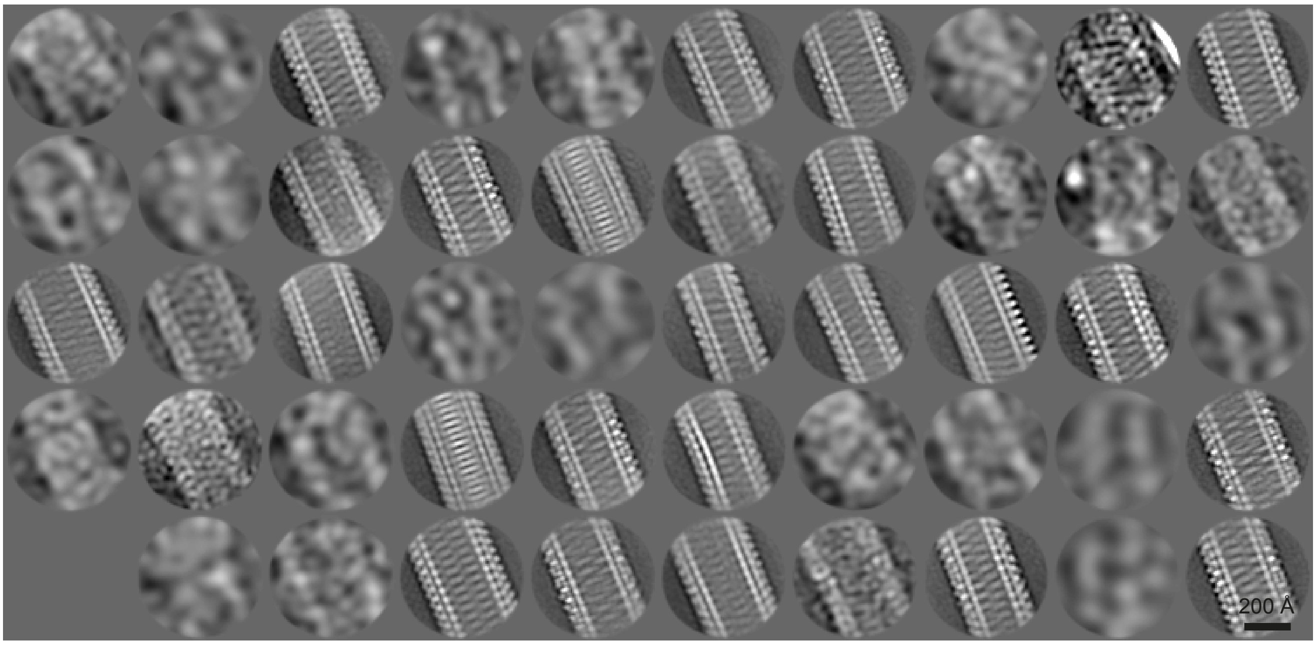

Figure S4

(A) STEM image of N-BAR tubes and TMV internal control. Boxes show examples of the MPL (mass per length) measurements (pink: N-BAR, blue: TMV). 20 μM of N-BAR were mixed with 720 μM of vesicles directly before the STEM sample preparation and measurement. (B) The distribution of N-BAR shows a MPL of 28±3 kDa/Å (N=148), while TMV shows 13±0.7 kDa/Å (N=66). The MPL was measured as described in Methods.

Supplementary Fig. S4

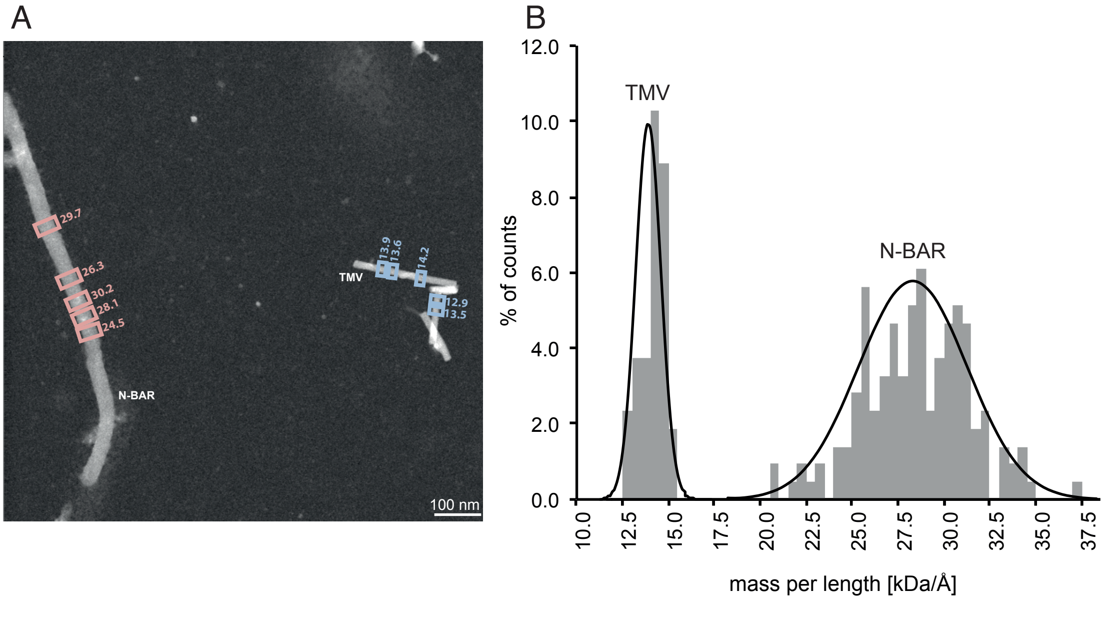

Figure S5

Quality control information of five selected classes of N-BAR mediated tubes. First column: Class averages. Second column: averaged power spectra of classified images (left) and the reprojections of the 3D reconstructions (right). Third column: Reprojections of 3D reconstructions. Fourth column: Density profile of the reprojection of the 3D reconstruction, revealing the tube radius. The profiles show three peaks from the tube center: inner leaflet, outer leaflet and the attached BAR protein densities. Fifth column: Fourier Shell Correlation profile. The resolutions were calculated to be 10.3, 11.2, 10.9, 10.9, 12.1 Å (top to bottom) at the FSC=0.5 cutoff criterion.

Supplementary Fig. S5

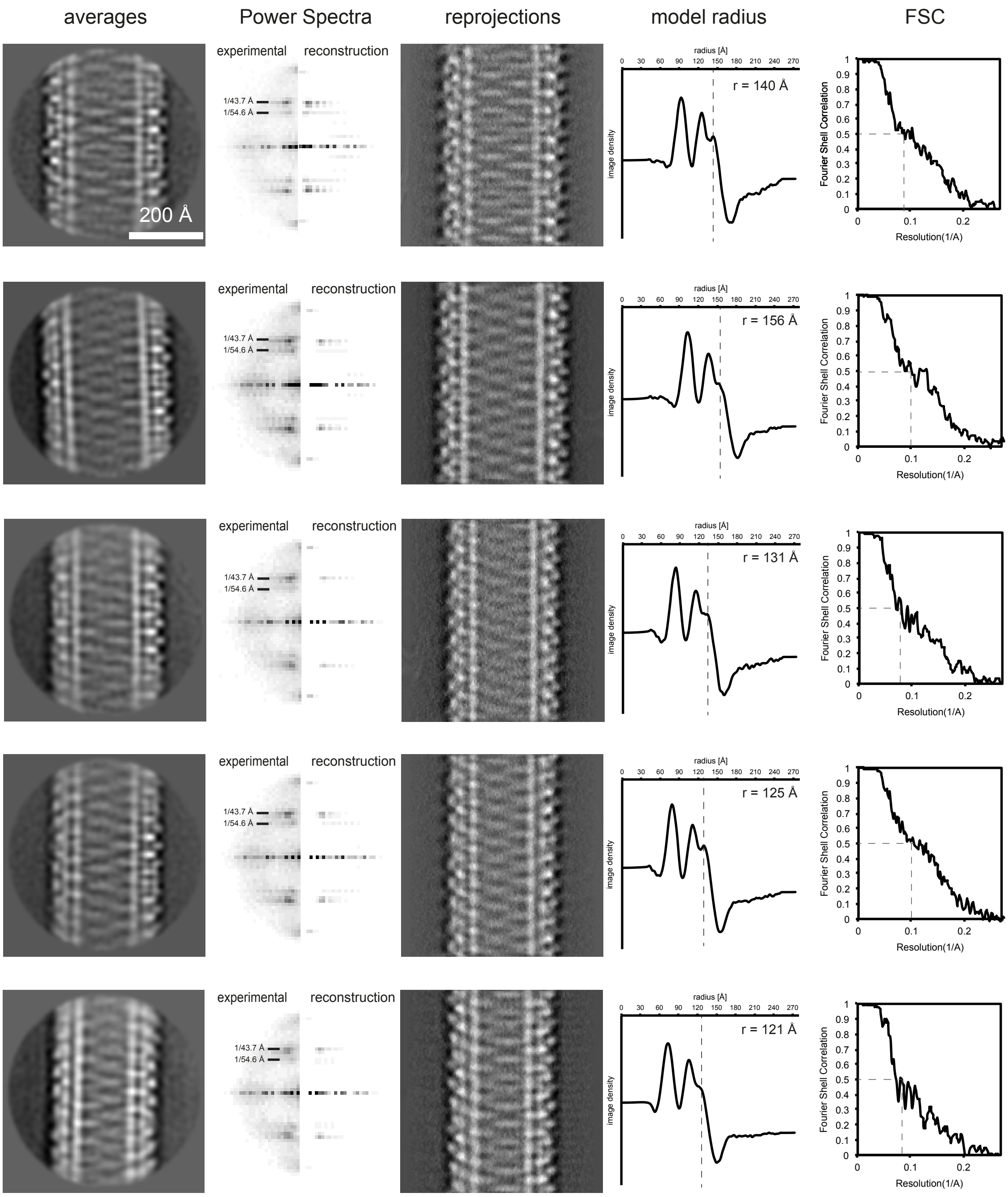

Figure S6

3D reconstructions of tubes with various sizes showing different arrangements of the BAR lattice packings. (A) The wider tube r = 156 Å reveals a slightly less tight BAR unit packing. The narrower tubes with (B) r = 131 Å, (C) r = 125 Å and (D) r = 121 Å show that the BAR units were rotated along the crescent dimer axis. For the fitting the amphiphysin BAR crystal structure (PDB: 1URU) was used.

Supplementary Fig. S6

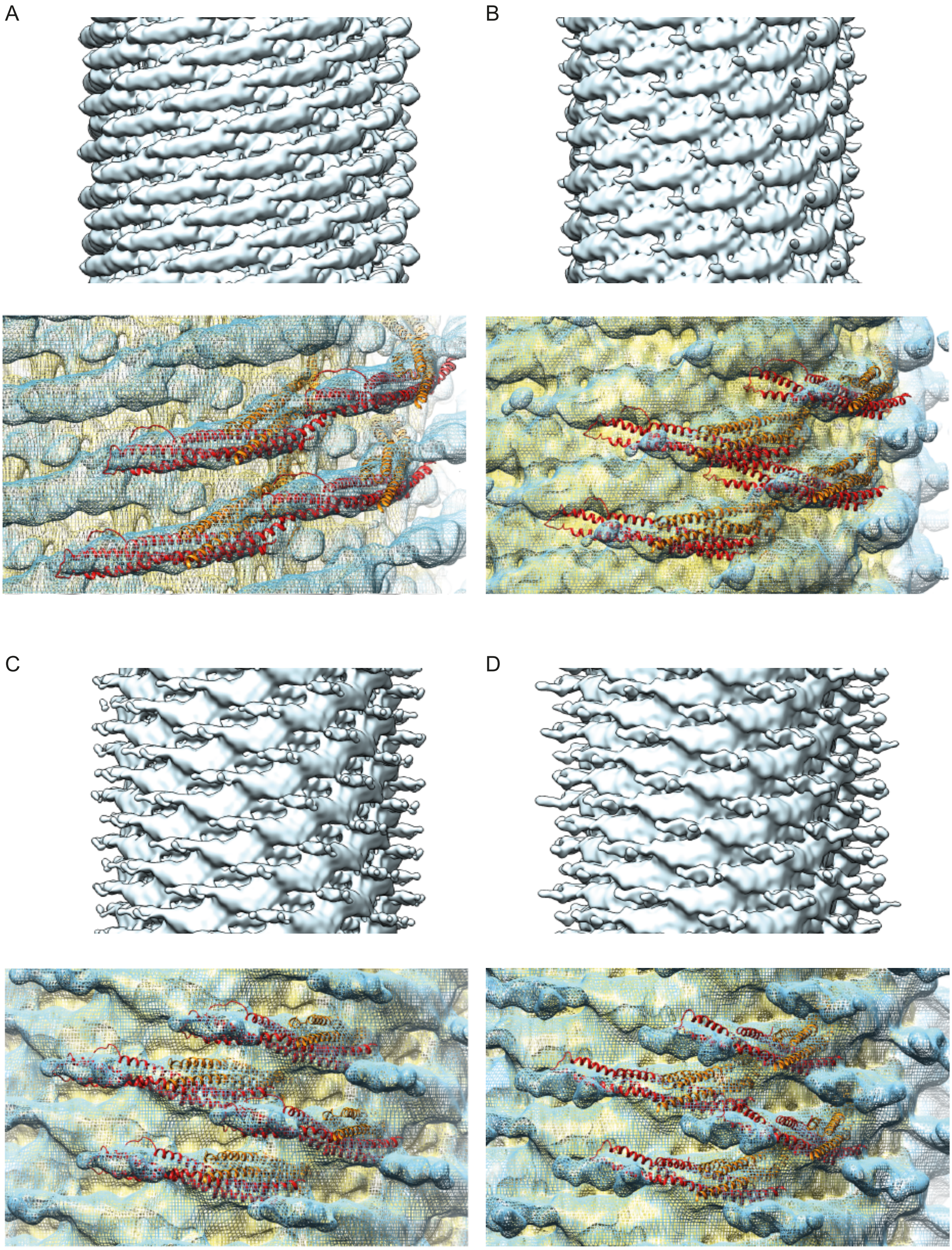

Figure S7

Membrane tubulation occurring by N-BAR-N-BAR-delta-tip heterodimer (left) and N-BAR-delta-tip homodimer (right). Tubulation of 720 μM fluorescently labeled vesicles mediated by 6 μM of the proteins observed by fluorescence light microscopy (top) and corresponding negative-stain EM observations (middle). v: examples of vesicles and t: examples of tubes. Bottom: Critical concentration measurements. Both protein fragments show tubulation of vesicles. The critical concentrations where no tubulation is happening anymore (guided with flat lines) were 0.2 μM for N-BAR-N-BAR-delta-tip and 0.7 μM for N-BAR-delta-tip. The measurement was done with the same mixture as in the fluorescence microscopy without labeled lipid.

Supplementary Fig. S7

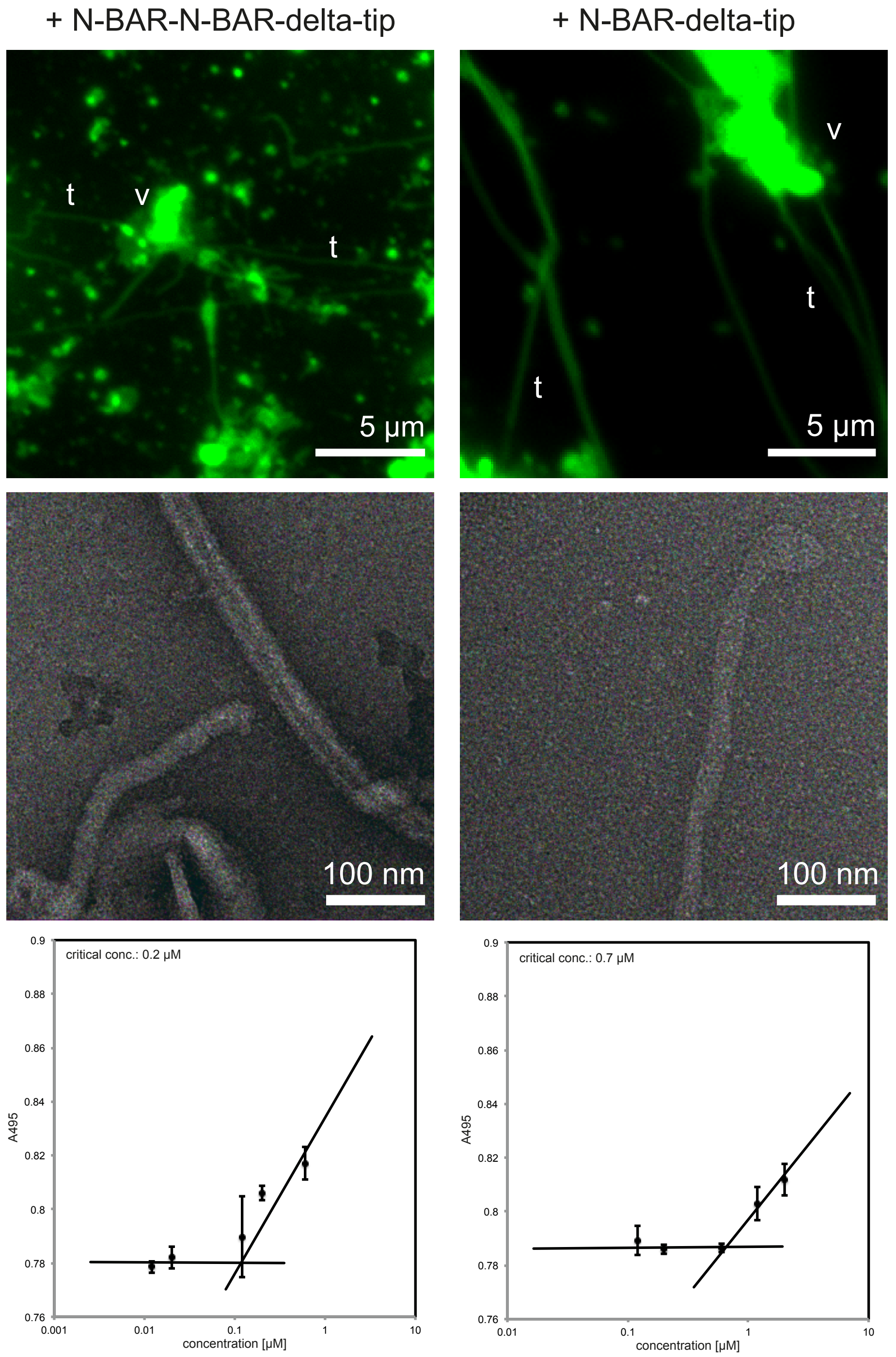

Supplement: Supplementary Information [file srep15452-s1.pdf]
